# First person – Sandeep Basu

**DOI:** 10.1242/dmm.049487

**Published:** 2022-03-28

## Abstract

First Person is a series of interviews with the first authors of a selection of papers published in Disease Models & Mechanisms, helping early-career researchers promote themselves alongside their papers. Sandeep Basu is first author on ‘ Latent TGFβ-binding proteins 1 and 3 protect the larval zebrafish outflow tract from aneurysmal dilatation’, published in DMM. Sandeep is a postdoctoral research fellow in the lab of C. Geoffrey Burns and Caroline E. Burns at Boston Children's Hospital, Harvard Medical School, investigating the modeling of cardiovascular diseases in zebrafish, drug discovery and deciphering molecular pathways in these contexts. Currently, he is investigating the molecular pathogenesis of a cardiovascular disease–thoracic aortic aneurysm and performing small-molecule screening to suppress the disease phenotype.

## How would you explain the main findings of your paper to non-scientific family and friends?

Aortic aneurysms are localized enlargements of the aorta, the largest artery in the human body, which supplies oxygenated blood from the heart to other organs. Owing to genetic or non-genetic risk factors, a segment of the aortic wall becomes weakened and, as a result, the aortic diameter begins expanding. When occurring in the chest cavity, this is called a thoracic aortic aneurysm (TAA). Uncomplicated TAAs are largely asymptomatic, making them difficult to detect. When early detection does occur, incidentally or as part of a syndrome, affected individuals are prescribed blood-pressure-lowering medications to slow disease progression. However, TAAs typically continue to enlarge until surgical correction is recommended to prevent acute aortic dissection, which is a tear in the inner cellular lining of the aneurysm. Dissections can restrict blood flow to the body or cause aortic rupture, both of which are life-threatening medical emergencies with high mortality. Even though current therapies are thought to slow disease progression, an unmet clinical need exists for medications that more effectively slow down or prevent disease progression and reduce the associated risk of dissection. Zebrafish have high genetic similarities to humans and are an excellent vertebrate model system for human disease modelling and drug discovery. However, animal models of TAA are largely restricted to genetically engineered mice. In this study, we created a novel zebrafish model of TAA. We knocked out two genes, *latent TGFb binding protein 1* and *2* (*ltbp1* and *ltbp3*, respectively), and observed that mutant zebrafish suffer from rapid and dramatic aneurysm of the zebrafish aorta in a segment analogous to where TAAs commonly occur in humans. Furthermore, we discovered that several cellular and molecular hallmarks of TAAs are recapitulated in our zebrafish model. Together, our data demonstrate that the unique advantages of zebrafish can now be leveraged to interrogate thoracic aneurysmal disease and screen for new therapeutic agents.

“The ongoing debate centers on whether canonical TGFβ signaling is protective or pathogenic for TAAs.”

**Figure:**
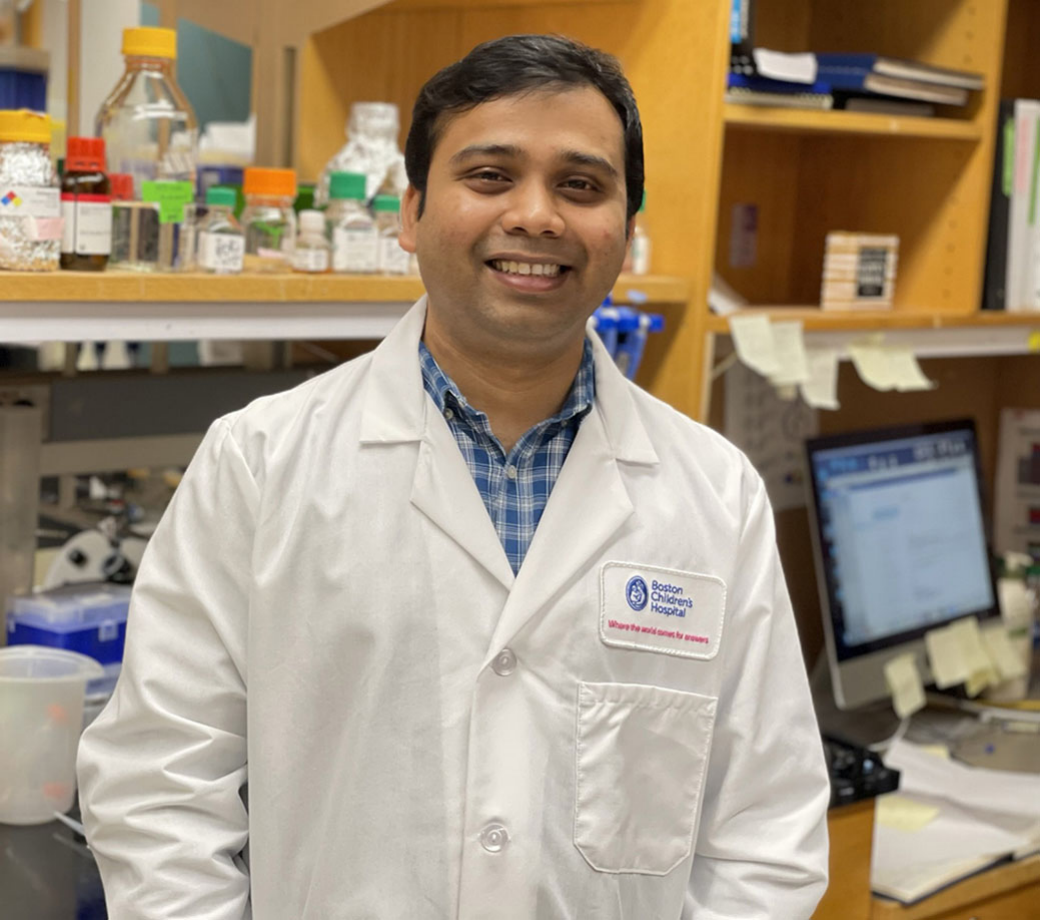
Sandeep Basu

**Figure:**
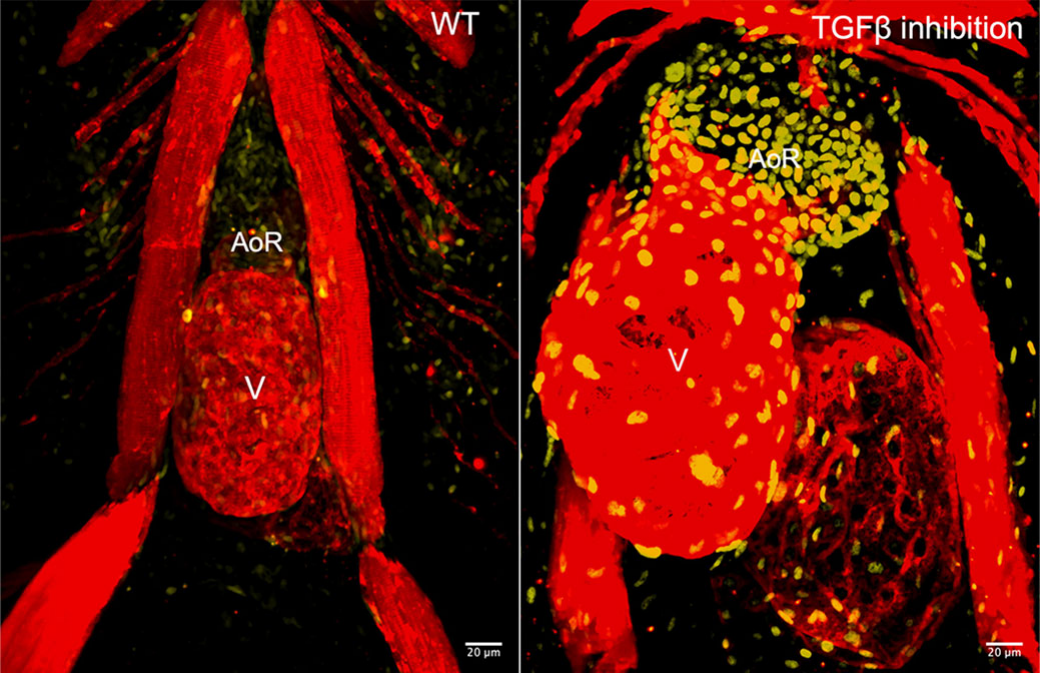
Inhibition of TGFβ signaling causes aortic root (AoR) aneurysm, ventricular (V) dilation and paradoxical hyperactivation of TGFβ signaling in zebrafish (yellow).

## What are the potential implications of these results for your field of research?

Beyond helping to establish zebrafish as a relevant model organism for TAA research and drug discovery, our results also provide some insight into the role played by TGFβ signaling in syndromic TAA pathogenesis, which has been controversial in the field for many years. The ongoing debate centers on whether canonical TGFβ signaling is protective or pathogenic for TAAs. Specifically, aneurysm tissue from patients and mouse models of TAAs display evidence for elevated TGFβ signaling but the causal mutations are thought to impair signaling, which has created a paradox. This paradox is recapitulated in our zebrafish model. Specifically, we observed that inhibition of TGFβ signaling is sufficient to induce aneurysm in zebrafish, demonstrating that baseline TGFβ signaling is protective. However, the distended aortic walls of the same animals exhibit evidence of TGFβ hyperactivation. Importantly, we were able to show that aneurysmal distention precedes TGFβ hyperactivation, suggesting that TGFβ hyperactivation is a downstream and secondary consequence of TGFβ inhibition or aneurysm itself rather than a primary driver of the disease phenotype. While this alone does not solve the paradox because the molecular mechanisms underlying this phenomenon are largely unknown, it supports the notion that TGFβ signaling can be protective rather than pathogenic. This could have implications for how we think about treating the disease.

## What are the main advantages and drawbacks of the model system you have used as it relates to the disease you are investigating?

Zebrafish embryos are small and transparent, and available in large numbers, attributes that make them suitable for small-molecule screening in a microwell format. We recently initiated a screen to identify chemical activities that suppress the TAA phenotype in our zebrafish model. It would be hard to imagine doing a similar screen with genetically engineered mouse models of TAA. One potential drawback is that aneurysm emerges in very young animals over the course of 48 h, whereas TAAs generally affect older animals and progress much more slowly in mouse models and humans. For screening purposes, this is an advantage, but a small molecule that suppresses a rapidly emerging phenotype in an immature zebrafish might not show the same benefit over the longer term in older mammals.

“ [...] our mutant animals also develop a dilated ventricle, a phenotype not typically observed in people affected by TAAs.”

## What has surprised you the most while conducting your research?

In addition to the aneurysm phenotype, our mutant animals also develop a dilated ventricle, a phenotype not typically observed in people affected by TAAs. Because the genes we knocked out are not expressed in the ventricle, we believe that ventricular dilation results from altered hemodynamics caused by aneurysm. Investigating the mechanism by which the ventricle dilates in response to aneurysm will be an interesting avenue of investigation moving forward.

## Describe what you think is the most significant challenge impacting your research at this time and how will this be addressed over the next 10 years?

Human genetics and mouse research have strongly implicated dysregulation of TGFβ signaling in the molecular pathogenesis of syndromic TAAs. However, as already mentioned, there is an ongoing controversy regarding whether high or low TGFβ signaling initiates the disease. Even though several lines of evidence suggest that low TGFβ drives disease pathogenesis, aneurysm tissue from humans, mice and now zebrafish display evidence for hyperactivated TGFβ signaling, leaving open the possibility that TGFβ hyperactivation also contributes to disease progression. Deciphering how low TGFβ signaling can ultimately lead to compensatory hyperactivation of the pathway will shed light on the ongoing controversy. This insight could also help to address the other challenge in the field, i.e. the need for more-effective therapies for preventing or even reversing aneurysm progression. Ongoing studies using model organisms, such as zebrafish, mouse and human cells, including patient-derived samples, as well as computational studies, will address these challenges. The hope is that our studies in zebrafish, including small-molecule screening, will provide valuable contributions to the field.

## What changes do you think could improve the professional lives of early-career scientists?

With the years of research experience of my own and all my peers in academia, I have observed huge variations regarding research training, number of collaborations and publication policies between the different institutes and laboratories that train postdoctoral fellows. However, the recruitment criteria for new principal investigators are very similar across the globe. They include high-profile publications, a track record of independent funding and a promising research plan. For instance, a postdoctoral fellow in a lab where projects are done in a highly collaborative manner are likely to have more publications compared to someone who single-handedly drives a project, resulting in fewer total publications. When it comes to faculty recruitment, many fellows might be less competitive because of the nature of the work performed during their postdoctoral fellowship, rather than their true scientific potential. In my opinion, a more normalized procedure to evaluate candidates should be designed. I also believe there should be standardized and formal training for both faculty and fellows on what is needed to make a smooth transition from postdoc to independent scientist in academia.

## What's next for you?

Following my postdoctoral fellowship, I would like to set up my independent research laboratory, preferably in my home country of India. I intend to use state-of-the-art molecular-genetic and chemical-genetic approaches to investigate cardiovascular disease pathogenesis and identify novel therapeutic interventions, primarily using zebrafish as a model system.
